# A multicenter randomized trial to improve family clinical note access and outcomes for hospitalized children: The Bedside Notes study protocol

**DOI:** 10.1002/jhm.70155

**Published:** 2025-08-21

**Authors:** Casey O’Hare, Amanda K. Gatewood, Jennifer Baird, Roger Brown, Ryan J. Coller, Arti Desai, Anna Egan, Danielle Gerber, Troy McGuire, Kristina Devi Singh-Verdeflor, Catherine Arnott Smith, George Angelos Verdelis, Gemma Warner, Sarah Wong, Michelle M. Kelly

**Affiliations:** 1 University of Wisconsin - Madison, Madison, Wisconsin, USA; 2 Children′s Hospital Los Angeles, Los Angeles, California, USA; 3 University of Washington School of Medicine, Seattle, Washington, USA

## Abstract

**Introduction:**

The 2021 Cures Act mandates caregiver access to their child′s medical notes with few exceptions, yet fewer than 10% access notes during hospitalization. Caregiver review of real-time notes facilitates identification of safety concerns and may enhance patient safety in pediatric hospitals. This trial will evaluate the Bedside Notes intervention—a multifaceted approach to enhance real-time access to inpatient notes—and its effects on caregiver activation, hospital experience, reporting of safety concerns found in notes, and anxiety.

**Methods:**

This 5-year, multisite randomized controlled trial will enroll 600 English and Spanish-speaking caregivers of hospitalized children ≤11 years old and 30 hospital staff across three hospitals. Caregivers will be randomized to usual care or the Bedside Notes intervention, which includes real-time inpatient note access via a bedside tablet, a caregiver orientation video, and a glossary of terms commonly found in notes. Our primary outcome is note access; secondary outcomes are caregiver activation, hospital experience, safety concerns, and anxiety, measured through electronic health record audits, surveys, and interviews.

**Discussion:**

We hypothesize that Bedside Notes will significantly improve caregiver note access, activation, hospital experience, and safety concern reporting without increasing caregiver anxiety. This study will also identify barriers and facilitators to accessing inpatient notes and inform scalable implementation strategies for caregiver engagement in hospital safety. Findings will advance efforts to reduce errors and improve family-centered care in pediatric hospital settings.

## INTRODUCTION

Medical errors affect up to one-third of hospitalized children,^1,2^ with harm occurring at three times the rate seen in adults.^3^ This increased risk stems in part from children′s reliance on both clinicians and caregivers to exchange critical health information and to identify and intercept safety concerns before harm occurs.^4^ However, the hospital environment poses significant challenges to effective communication. Caregivers—often exhausted and stressed—typically meet clinicians for the first time during morning rounds and must make rapid, high-stakes decisions. These brief interactions often involve dense verbal information that is rarely supported by accessible written or visual materials. When written materials are provided, they often lack plain language^5^ and are not translated for caregivers who speak languages other than English.^6^ These barriers limit caregivers′ ability to identify safety concerns and prevent harm, hindering efforts to meaningfully engage them in patient safety.

Providing caregivers with access to clinical notes, which outline their child′s diagnoses and treatment plans, has emerged as a promising strategy to improve their engagement and safety outcomes.^7^ However, despite the 21st Century Cures Act mandate for note-sharing,^8^ caregiver access to notes remains low. For example, local data at the University of Wisconsin-Madison (UW) in 2022 demonstrated that 99.6% of inpatient notes were shared, yet fewer than 10% were accessed by at least one caregiver. Multiple barriers to access remain, including but not limited to the need for a personal device, internet access, patient and caregiver proxy portal enrollment, and caregiver understanding how to navigate and interpret notes.^9^

The Bedside Notes intervention was developed to enhance caregiver access to inpatient notes through (1) real-time access via a bedside tablet pre-registered to the child′s patient portal; (2) an orientation video explaining how to access notes, when they become available, and how to raise concerns; and (3) a glossary of commonly used terms found in notes in both English and Spanish. In a pilot study, 96% of caregivers accessed at least two notes—a 10-fold increase over standard access. Among those who reviewed notes, 20% identified potential safety concerns, 60% of which were confirmed safety issues. Caregivers who accessed notes reported increased activation and decreased anxiety from admission to discharge and improved hospital experience.^10^

This study will evaluate the Bedside Notes intervention in a multisite randomized controlled trial, assessing its impact on caregiver note access, activation, hospital experience, safety concern reporting, and anxiety. We will engage a diverse caregiver population and incorporate feedback from national stakeholders to ensure broad applicability. This will be the first multisite study on inpatient note-sharing for hospitalized children and the first trial assessing its impact on caregiver-reported outcomes. By improving caregiver access to inpatient notes, this study aims to strengthen family engagement in pediatric inpatient safety, inform best practices for integrating health information technology (IT) into hospital care, and enhance communication between caregivers and pediatric healthcare providers.

## METHODS

### Study design

In this multicenter randomized controlled trial, our primary hypothesis is that caregivers in the intervention arm will have higher odds of accessing inpatient notes (defined as opening at least two available notes during hospitalization) than those in the usual care group. Additionally, we hypothesize that, compared with usual care, caregivers in the intervention group will report increased activation in their child′s care from admission to discharge,^11^ improved hospital experience,^12^ and higher rates of safety concerns found in notes^13^ without an increase in anxiety.^14^ Participant enrollment will take place between approximately April 2025 and December 2028 across three sites. Each caregiver will be followed for the duration of their child′s hospital stay.

### Conceptual framework

**FIGURE 1:**
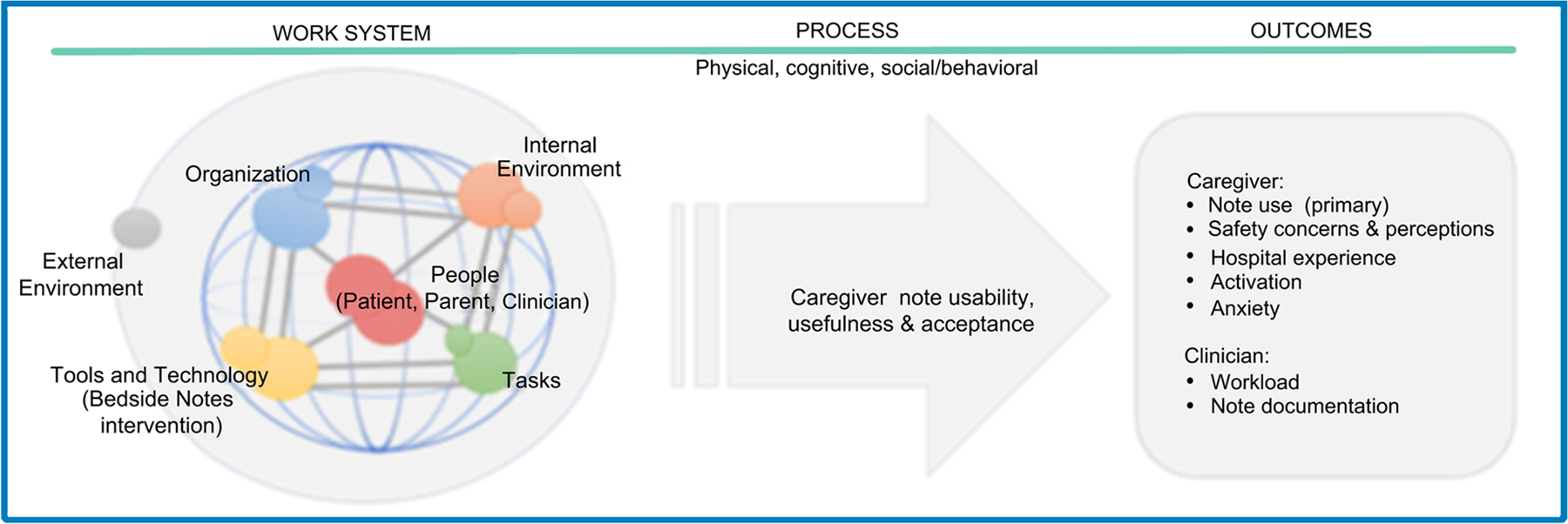
Conceptual framework for sharing inpatient notes with caregivers of hospitalized children, adapted from the Systems Engineering for Patient Safety (SEIPS) 2.0 model.^16^

The evaluation of the Bedside Notes intervention will be grounded in a sociotechnical systems approach, recognizing that the design, implementation, and evaluation of health IT are as critical to success as the technology itself. Many health IT interventions fail when viewed as purely technological solutions (e.g., simply providing a tablet or enabling note-sharing) without addressing human and organizational factors.^15^ To mitigate these risks, this study is guided by the Systems Engineering Initiative for Patient Safety (SEIPS 2.0) framework, a well-established model that describes the work system and environmental factors that shape how an intervention functions in real-world settings.^16^ This framework will guide a concurrent evaluation of the intervention′s impact and implementation (Figure 1). A mixed-methods, convergent parallel design will integrate quantitative and qualitative data from caregivers and hospital staff to assess usability, perceived benefits, and challenges.

Caregiver advisors were involved as stakeholders in the design of the intervention,^17^ the pilot study,^10,18^ and this trial. Two lived-experience partners refined the intervention′s educational materials and continue to valuably contribute to study design and execution as members of the study′s steering committee.

By applying this comprehensive sociotechnical approach and directly partnering with caregiver advisors, this study will inform scalability, sustainability, and optimization strategies for the Bedside Notes intervention, ensuring that the anticipated benefits are maximized while minimizing unintended consequences.

### Setting and population

The study will be conducted at three children′s hospitals: (1) the American Family Children′s Hospital of the UW, (2) Seattle Children′s Hospital, and (3) Children′s Hospital Los Angeles (CHLA). These sites were chosen due to established collaborations, diverse patient populations, and representation of multiple electronic health record (EHR) platforms. The study will enroll approximately 600 caregivers of hospitalized children, evenly distributed across sites (200 per site). Additionally, 30 hospital staff (five physicians and five nurses at each site) will be enrolled to assess staff perspectives on the intervention. Data collection will be complete at each site once 200 caregivers and 10 staff have completed study activities.

### Eligibility criteria

Each pediatric patient will be represented by one enrolled caregiver. Caregiver participants must be the parent or legal guardian of a child who is ≤11 years old who is admitted to a pediatric service. This age limit is based on legal restrictions related to caregiver access to adolescent health information. Eligible caregivers must be fluent in English and/or Spanish, be at least 18 years old, and have not previously participated in the study. The hospitalized child must have an anticipated overnight hospital stay to ensure sufficient exposure to the intervention. Caregivers will be excluded if there are concerns about guardianship, suspected abuse, or neglect or they are already enrolled in another inpatient study.

Hospital staff participants must be at least 18 years old and employed as a physician or nurse at one of the participating hospitals. To be eligible, the staff member must have provided direct care to a child in the intervention group on the day of discharge.

### Participant involvement

Each day, research coordinators at each site will screen the EHR to identify eligible caregiver participants. Once identified, the coordinator will confirm eligibility and consult with the patient′s nurse to determine appropriate timing for enrollment. If eligible, the coordinator will approach the caregiver in person to introduce the study, provide detailed information about participation, and address any questions. Caregivers who express interest will complete a signed consent form (Appendix S1) and an electronic enrollment survey (Appendix S2). Participants will then be randomized to either the intervention group (Bedside Notes), receiving real-time inpatient note access, or the usual care group, receiving standard patient portal access. Each participant will be provided a copy of the signed consent form, including the principal investigator′s contact information in case they wish to withdraw from the study.

Hospital staff participants will be identified via EHR review based on which physicians and nurses provided care to children in the intervention group on the day of discharge. These staff members will be invited to participate in a 30-min interview and complete a 5-min demographic survey. Verbal consent will be obtained before participation.

### Interventions

Participants will be randomized to one of two study conditions:

#### Usual care

Caregivers will receive a handout (available in English and Spanish) with standard hospital information on how to register for the patient portal and access clinical notes from a personal device. Research staff will not provide further assistance with portal enrollment or note access.

#### Bedside notes intervention

Caregivers will receive real-time inpatient note access via a bedside tablet linked to the hospital′s patient portal (Epic at UW and Seattle Children′s, Oracle at CHLA). They will also view a 90-s orientation video (available in English and Spanish) explaining the purpose of notes, how to access them, and who to contact with questions or concerns. Additionally, a glossary of commonly used terms in both English and Spanish will be provided.

On the day of discharge, all caregivers will complete a final survey (Appendix S3). A subset of caregivers from the intervention group will be invited for a 30-min interview to discuss their experiences with Bedside Notes.

### Randomization and blinding

**FIGURE 2:**
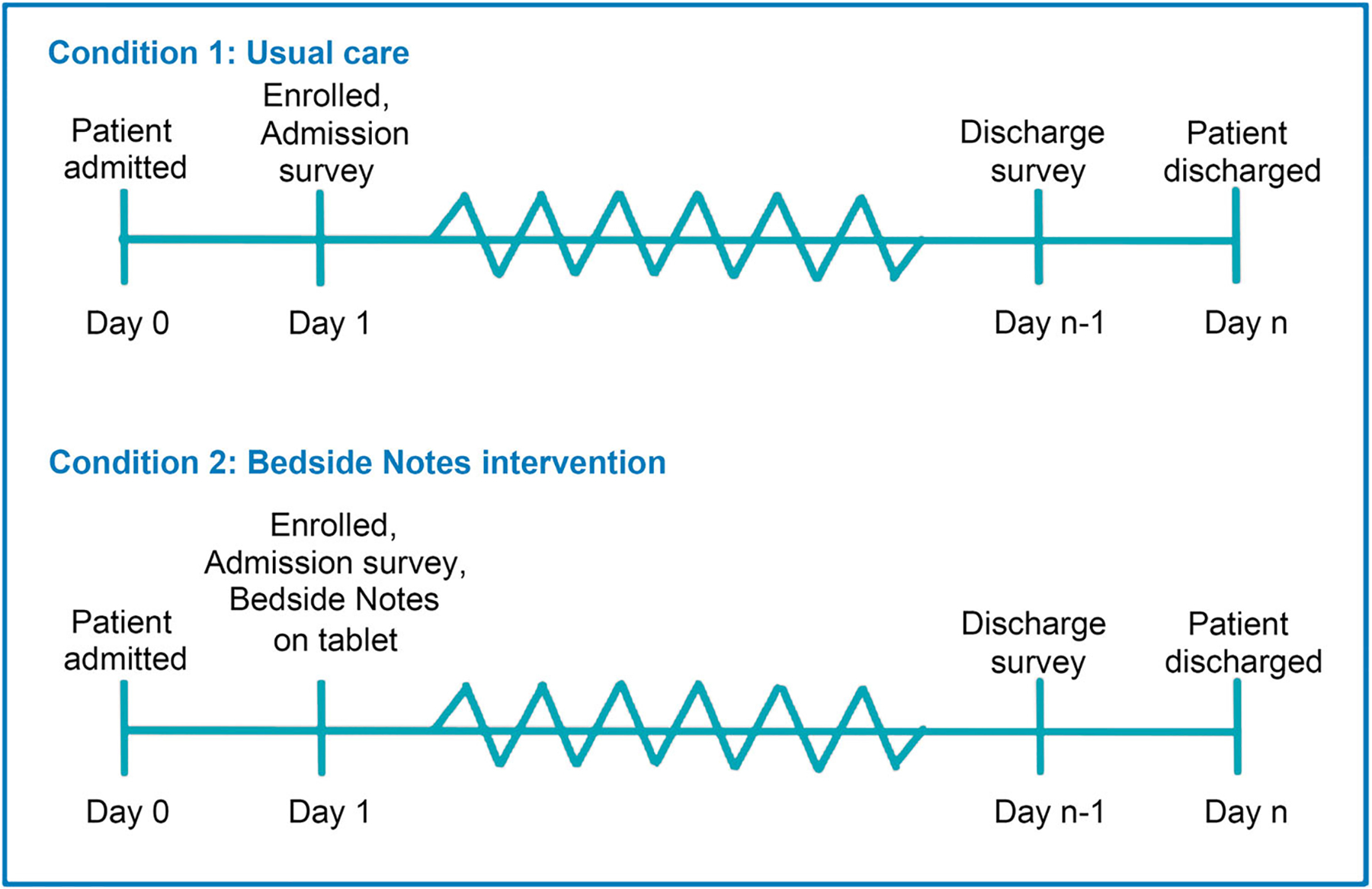
Randomization at the patient level.

Randomization will occur at the patient level in research electronic data capture (REDCap) and will be stratified by site. Caregiver participants will be randomized in a 1:1 ratio by permuted block randomization using varied block sizes of two and four (Figure 2). Given the nature of the intervention, blinding participants is not feasible. Staff may also become aware of the assignment if caregivers reference note content accessed on the tablets. However, researchers conducting outcome assessments and data analysis will remain blinded to group allocation.

### Outcomes and covariates

Outcome definitions, sources, and measurement methods are detailed in Table 1. The primary outcome is note access, defined as the proportion of caregivers who open at least two inpatient notes during their child′s hospitalization. This will be assessed using EHR audit reports, which capture the type, frequency, and proportion of notes written and shared by the healthcare team and accessed by caregivers. These audit reports have been used in prior studies to evaluate inpatient portal use and note access.^10,23,24^

Secondary outcomes include:

#### Caregiver activation

The Parent-Patient Activation Measure (P-PAM)^11^ will assess caregivers′ skills and confidence in managing their child′s healthcare. Adapted from the Patient Activation Measure, the P-PAM includes 13-item Likert-style items, scored on a 0–100 scale, with higher scores indicating greater activation. It has demonstrated acceptable validity and reliability and will be administered at admission and discharge in English and Spanish.

#### Hospital experience

Caregivers will complete the Child Hospital Consumer Assessment of Healthcare Providers and Systems (HCAHPS) survey^12^ at discharge. This validated tool includes Likert-style items rated on a 5-point scale. We will assess overall hospital rating, willingness to recommend, and whether the hospital helped caregivers report concerns. It will be available in English and Spanish.

#### Safety concern reporting

Safety concerns will be assessed using a modified version of the OpenNotes safety concern reporting tool, a 9-item questionnaire adapted for the pediatric inpatient setting. Available in English and Spanish, this tool captures caregiver-reported safety concerns found in their child′s clinical notes.^13^ Reported concerns will be reviewed and categorized by the principal investigator and site PIs as definite safety issues, possible safety issues, or other, and further classified by type (e.g., medication-related, physical exam findings). A chart review will determine whether each confirmed issue resulted in changes to the medical record or patient care.

#### Caregiver anxiety

The State-Trait Anxiety Inventory (STAI) Form Y^14^ will assess caregiver anxiety at admission and discharge. This 40-item, Likert-style instrument assessed both state and trait anxiety on a scale from 20 to 80, with higher scores indicating greater anxiety. The STAI has been validated and shown to be responsive to health interventions.^25^ It will be available in English and Spanish.

Covariates will be collected on admission and include factors known or hypothesized to be associated with caregiver engagement and/or study outcomes. These include demographic and clinical characteristics (age/sex/gender, child relationship, race/ethnicity, education, income, health status, hospital stays, experience with chronic illness, and previous note access), language proficiency,^26^ health and digital literacy,^19,21,27^ and medical complexity.^22^

### Interviews

To gain deeper insights into the implementation of the Bedside Notes intervention, we will conduct semi-structured interviews of both caregivers and hospital staff. Trained research coordinators will facilitate the interviews to minimize bias and encourage open, candid responses. Interviews will follow a semi-structured script with openended questions, initially exploring participants′ experiences, perceived benefits, and challenges of the intervention. Probing questions will explore work system and process elements guided by the SEIPS 2.0 model.^16^

At the end of the staff interview, study coordinators will administer a survey assessing participants′ experiences with the Bedside Notes intervention. The survey will include validated SEIPS items measuring usability, usefulness, acceptance, and perceived impact on workload.^28^ Additional items adapted from the Ambulatory OpenNotes survey^13^ will assess satisfaction with note sharing and access, as well as perceptions of how notes influence caregiver understanding, care quality, and documentation. Surveys will also collect demographic and clinical characteristics for staff (e.g., age and years in practice).

Each ~30-min interview will be audio-recorded and conducted either at the child′s bedside or in a private hospital conference room, based on participant preference. Spanish-speaking participants will be interviewed by a bilingual coordinator or with the assistance of a professional interpreter. Staff interviews will be conducted in person or remotely via secure Zoom. A professional transcription service will transcribe interviews in English and Spanish and translate when necessary. Study coordinators will review transcripts for completeness, clarify unclear text, and remove any inadvertently included identifying details. Transcribed data will be securely uploaded to Dedoose to facilitate qualitative analysis.

### Data collection and security

Study data will be collected from three primary sources: (1) the EHR, (2) quantitative surveys administered at enrollment and discharge, and (3) qualitative interviews with caregivers and hospital staff (Table 2).

EHR data will capture inpatient note access, while caregiver and hospital staff perspectives will be gathered through surveys and interviews. All participant data, including screening logs, consent forms, survey responses, and interview transcripts, will be securely stored in REDCap and a restricted-access cloud database, ensuring compliance with HIPAA and institutional data security protocols. Deidentified data will be maintained in a separate external REDCap database accessible to study personnel across all sites. Audio-recordings from interviews will be permanently deleted following transcription to protect participant confidentiality. Study documentation, including protocols, standard operating procedures, and recruitment materials, will be stored in a shared but restricted-access cloud drive to facilitate coordination among study sites.

Research coordinators and the principal investigator will oversee data monitoring. Coordinators will conduct routine audits to ensure the completeness and accuracy of data collection. Any adverse events will be reported to the site principal investigator within 24 h of notification. The principal investigator will follow the site-specific adverse event protocol, including notifying the institutional review board (IRB) when necessary.

### Sample size considerations

The study is powered to detect differences in both note access and safety concern reporting between intervention and control groups. For note access, baseline data show that 9% of caregivers in usual care accessed two or more notes, compared with 96% of caregivers in the pilot intervention group. For this trial, we conservatively estimate that 50% of caregivers in the intervention group will access two or more notes. To detect this difference with 80% power at the two-sided 5% significance level, a total of 50 caregivers (25 per arm) is required. For safety concerns, prior research indicates that 1.2% of caregivers in the usual care group and 6% in the intervention group report a confirmed safety concern.^13^ To detect this difference with 80% power at the two-sided 5% significance level, at least 502 caregivers (251 per arm) are needed.

To account for potential attrition, the study will enroll 600 caregivers total (200 per site; 300 per arm). Each site is expected to enroll two to three caregivers per week, a pace deemed feasible given annual pediatric admission volumes of 1500 at UW, 5500 at Seattle Children′s, and 5200 at CHLA.

### Statistical analyses

The primary analysis will follow an intent-to-treat approach, including all eligible participants who were randomized. Continuous variables will be summarized using the number of observations, mean, standard deviation, coefficient of variation, median, and range, as appropriate. Categorical variables will be summarized using frequencies and percentages. The number and percentage of caregivers screened, randomized, and reasons for screening failure or discontinuation will be summarized in tabular form. Baseline characteristics will be summarized and compared between study arms using two-sample *t*-tests or nonparametric Wilcoxon rank-sum tests (nonparametric) for continuous variables or χ^2^ or Fisher′s exact tests for categorical variables.

The primary outcome is note access, defined as the proportion of caregivers who open two or more available inpatient notes during their child′s hospital stay. Note access rates will be compared between study arms using a stratified Mantel–Haenszel test, with study site as the stratification factor to account for the stratified randomization. The secondary outcome is defined as the proportion of caregivers who report at least one safety concern. This outcome will also be analyzed using the stratified Mantel–Haenszel test.

For other instrument-based outcomes, including caregiver activation, hospital experience, and anxiety, results will be summarized by study arm using means, standard deviations, medians, and ranges. Group comparisons between will be conducted using stratified *t*-test or Wilcoxon rank-sum tests (nonparametric). Exploratory subgroup analyses will assess differences by health literacy, language proficiency, digital literacy, and other demographic and clinical factors.

We will assess missing data patterns using Little′s test (missing completely at random) and Potthoff′s assessment (missing at random).^29^ If missing data are detected, we will select and implement a method of imputation based on observed patterns of missingness (e.g., simple imputation method, regression-based, multiple imputation). Withdrawn participants′ available data will be included in analyses. All analyses will be conducted using Stata Version 18.0.

### Qualitative analysis

We will use directed content analysis^30^ to analyze data from caregiver and staff interviews, guided by the SEIPS 2.0 model.^16^ Coding categories will include work system elements (e.g., External Environment, Technology/Tools, Tasks, Organization, Environment, Caregiver/Hospital Staff) and process elements (e.g., Note Sharing and Access). To ensure rigor and reduce disciplinary bias, a team of four researchers will conduct the analysis. At least two coders will independently review each transcript, then reconcile discrepancies through consensus discussion. If content does not fit within existing SEIPS 2.0 categories, it will be flagged for review and considered for new codes or subcomponents. The coding framework will be refined iteratively, with codebooks developed to capture key domains and illustrative quotes.

### Research ethics consideration and approval

The study was approved by the UW IRB in November 2024, with reliance agreements pending at the two partner sites. All modifications to the protocol will be submitted as amendments for IRB review and approval.

All study materials are written using plain language principles and translated into Spanish for bilingual participants. Interpreters will be available to assist with additional verbal communication if needed. All participant data will be securely stored in REDCap and a restricted-access cloud database, in compliance with HIPAA and institutional data security protocols.

Participants will receive an incentive for their involvement. Caregivers will receive a $20 gift card upon completing the discharge survey and an additional $20 gift card if they complete an interview. Hospital staff participants will receive a $20 gift card for completing their interview.

### Dissemination plan

Study findings will be shared through multiple channels to support broader implementation of inpatient note-sharing practices. Real-time analysis of caregiver-reported safety concerns will be disseminated to participating hospitals to information quality improvement efforts.

The study team will discuss key findings locally (e.g., with Patient and Family Advisory Councils, pediatric clinical teams) and nationally (e.g., through conferences and publications). Additionally, results will be reported to the Agency for Healthcare Research and Quality to inform broader health IT initiatives. The study is registered on ClinicalTrials.gov, where results will be updated in compliance with federal requirements.

## DISCUSSION

### Study implications

The overarching goal of this study is to enhance caregiver access to trusted health information to improve the safety of pediatric inpatient care. Specifically, this trial evaluates the impact of the Bedside Notes intervention on caregiver note access and safety-related outcomes. Based on preliminary research,^17,23,24,31–35^ we hypothesize that increasing caregiver access to inpatient notes will improve engagement, facilitate detection of safety concerns, and contribute to better hospital experiences for families of hospitalized children.

### Strengths and limitations

This study has several notable strengths. It leverages patient portals from two major EHR vendors—Epic Systems^36^ and Oracle^37^—allowing an assessment of interoperability across platforms. Additionally, it examines note-sharing across multiple hospital staff roles, including physicians, nurses, consultants, and ancillary providers, offering a comprehensive view of how caregivers interact with clinical documentation.

A key strength is the application of the SEIPS 2.0 sociotechnical framework^16^ to guide qualitative analysis. This well-established model facilitates systematic evaluation of how the intervention interacts with caregivers, clinicians, workflows, and the broader hospital environment, supporting the development of actional insights for future implementation of consumer-facing health IT interventions.

However, several limitations must be acknowledged. Self-reported outcomes, such as safety concerns and hospital experience, may be subject to recall bias or social desirability bias. Differences in caregiver engagement—driven by factors like digital literacy, prior portal use, or personal preference—may influence outcomes. While randomization should evenly distribute these characteristics across study arms, residual confounding cannot be ruled out. We will use statistical adjustments to address known covariates, but unmeasured factors such as clinician communication style or prior hospitalization experiences may still influence caregiver responses.

Implementation fidelity is another potential challenge. If clinicians do not consistently document timely or clear notes, caregivers may struggle to engage meaningfully with the content. Some staff may also view sharing notes with caregivers as an additional burden, which could influence how notes are written and shared across the sites. These effects are expected to occur across both study arms but may affect real-world scalability.

Generalizability of study results may also be limited by the setting: all three sites are large, urban pediatric hospitals with robust digital infrastructure and experience with patient portals. Findings may not translate directly to rural hospitals or settings with more limited resources.

Lastly, the study focuses on short-term hospitalization outcomes; the long-term impact of inpatient note access on caregiver behavior and safety outcomes after discharge remains unknown and an important area for future investigation.

## CONCLUSIONS

The Bedside Notes intervention represents a promising approach for improving caregiver access to clinical information during hospitalization and supporting implementation of the 2021st Cures Act. By enhancing transparency, fostering caregiver engagement, and enabling early identification of safety concerns, this intervention may advance broader efforts to promote family-centered care. Future research should explore expanding this model beyond pediatric inpatient settings to other populations and care environments, further advancing equitable access to health information and promoting patient safety more broadly.

## Supplementary Material

Appendix 1

Appendix 3

Appendix 2

Additional supporting information can be found online in the Supporting Information section at the end of this article.

## Figures and Tables

**TABLE 1:** Trial outcomes with definitions and sources.

Measure	Item	Source
Note access	Caregivers who open two or more available notes	Electronic health record
Activation	P-PAM^11^	Admission and discharge surveys
Experience	Child HCAHPS^12^	Discharge survey
Safety concerns	OpenNotes safety concern reporting tool^13^	Discharge survey
Anxiety	STAI inventory^14^	Admission and discharge surveys
Health literacy	BRIEF screener, short assessment of health literacy^19,20^	Admission survey
Language proficiency	Agency for Healthcare Quality and Safety Health Literacy Universal Precautions Toolkit^21^	Admission survey
Demographic & clinical characteristics	Digital literacy, education, income	Admission survey
Child’s chronic and complex illness	Chronic conditions classification system version 2^22^	Electronic health record

Abbreviations: BRIEF, Brief Health Literacy Screening Tool; HCAHPS, Hospital Consumer Assessment of Healthcare Providers and Systems; P-PAM, Parent-Patient Activation Measure; STAI, State-Trait Anxiety Inventory.

**TABLE 2:** Database descriptions.

Location	Accessible by	What information will be stored	Notes
Internal REDCap	Respective site study staff	Screening and enrolled participant information with personal health information, electronic health record review, Adverse event record	An internal REDCap database will be created for each study site. Only the study staff of the respective site will have access to the information stored here. The lead site will send templates for database creation. Once deidentified, all study staff will have access to the internal REDCap data.
External REDCap	All study staff	Deidentified screening information, Completed consent forms, Randomization, Survey responses, Deidentified interview transcriptions	All study staff of all sites will have access to the external REDCap database.
Shared restricted cloud storage	Respective site study staff	Interview recordings, Copies of completed consent forms, Audits	
Shared unrestricted cloud storage	All study staff	Study protocol, Study standard operating procedure, Document templates (consent form, surveys, information sheets, etc.), Interview guides, Recruitment scripts	

Abbreviation: REDCap, research electronic data capture.

## Data Availability

De-identified survey data, redacted interview transcripts, codebooks, and analytic code will be available from the corresponding author upon reasonable request and completion of a Data Use Agreement. Proprietary scale data, raw audio recordings, and unredacted transcripts will not be shared.
