# Supplementary material for: A multicenter randomized trial to improve family clinical note access and outcomes for hospitalized children: The Bedside Notes study protocol
Source: J Hosp Med. 2025 Aug 21;20(11):1256–64. doi: 10.1002/jhm.70155 (PMC12443148; doi:10.1002/jhm.70155)
Supplement: Supplementary file 1 — Appendix 1 ‐ Participant Consent Form. [file JHM-20-1256-s003.docx]

*Si desea ver esta encuesta en español, haga clic en el símbolo del globo terráqueo que se encuentra arriba en la esquina derecha.*

**University of Wisconsin-Madison**

**Consent to Participate in Research and**

**Authorization to Use Protected Health Information for Research**

______________________________________________________________________

## Study Title for Participants: Bedside Notes

## Formal Study Title: Bedside Notes: A multicenter trial to improve family clinical note access and outcomes for hospitalized children

## Lead Researcher:

Dr. Michelle Kelly

Phone: 608-262-7229

Email: [michelle.kelly@wisc.edu](mailto:michelle.kelly@wisc.edu)

## Institution: American Family Children’s Hospital, University of Wisconsin-Madison

______________________________________________________________________________

## Key Information

## The information in this section is to help you decide whether or not to be a part of this study. You can find more detailed information later on in this form.

## Why are researchers doing this study?

You are invited to join a study to help improve how parents can see their child’s medical notes while they are in the hospital. We want to find out if letting parents see these notes on a tablet (iPad) can help them feel more involved, talk better with doctors, and improve care.

You can take part because you are a parent or guardian of a child in one of these hospitals: American Family Children’s Hospital (WI), Children’s Hospital of Los Angeles (CA), or Seattle Children’s Hospital (WA).

## What will I need to do in this study?

The treatment you get will be chosen by chance; like flipping a coin. Neither you nor the study doctor will choose what group you get assigned to. You will have an equal chance of being placed in one of the following two groups:

- Group 1 (Intervention Group): If you are in this group, you will get access to the notes written by your child’s care team on an iPad while your child is in the hospital. You will also watch a short video explaining how to use the tablet to see the notes.
- Group 2 (Control Group): If you are in this group, you will not get access to the notes or the instructional video. You will receive the usual care at the hospital.

You will complete two short surveys: one today and one within 24 hours after your child leaves the hospital. Each survey takes about 5-10 minutes. After you finish the second survey, you will receive a $20 gift card.

Some people in Group 1 will be invited to do an optional interview to share their experiences with the notes. If chosen, this interview will take about 30 minutes, and you will get another $20 gift card.

**What are some reasons I might or might not want to be in this study?**

| **You may want to be in this study if you are:** | **You may NOT want to be in this study if you:** |
| --- | --- |
| - Interested in viewing your child’s medical notes. - Comfortable having researchers ask questions about you and your child. - Interested in contributing to scientific knowledge even though you won’t benefit directly from the study. | - Want to be in a study that might help improve your own health. - May not have time to complete study surveys. - Are not comfortable using technology. |

## Do I have to be in the study?

No, you don’t have to join this study. Being part of the research is your choice. If you decide not to participate, it won’t affect your or your child’s healthcare or any services you receive. There will be no penalty, and you or your child won’t lose any medical care or legal rights. You can ask as many questions as you like before making your decision.

## Detailed Information

## The following is more detailed information about this study.

## How is research different from health care?

When you take part in a research study, you are helping to answer a research question. Study surveys and interviews are not for your or your child’s health care.

## Who can I talk to about this study?

If you have questions, concerns, or complaints, or think that participating in the research has hurt you or your child, talk to the research team at [michelle.kelly@wisc.edu](mailto:michelle.kelly@wisc.edu).

If you have concerns about your rights as a research participant or have complaints about the research study or study team, call the confidential research compliance line at 1-833-652-2506. UW Staff not part of the study team will work with you to address concerns and assist in resolving any complaints.

## If I take part in the study, what will I do?

As part of the study, we will survey all participants. You will be given one survey the day that you enroll into the study, and another survey the day your child is discharged from the hospital. If we are unable to give you the second survey before you leave, we will send you the survey either by email or ask you survey questions over the phone. After we receive the discharge survey, you will be given a $20 gift card.

While your child is admitted, you will be assigned to one of two groups. If you’re in Group 1, the intervention group, you will get access to the notes written by your child’s care team on an iPad while your child is in the hospital. You will also watch a short video explaining how to use the tablet and see the notes. If you’re in Group 2, the control group, you will not get access to the notes or the instructional video. You will receive the usual care at the hospital.

Some participants in Group 1 will also be asked to participate in an interview. Not everyone will be chosen for an interview. If you are selected and take part in an interview, we will record video or audio of you to save your answers to the interview questions. These recordings will only be used for the study and will not appear in any papers or publications. A written copy of the recordings will be made for research purposes. After we complete the interview, you will be given another $20 gift card.

**Protected health information (PHI) used in this study**

Protected health information, also called PHI, is information about your or your child’s physical or mental health that includes names or other information that can identify you or your child, like their date of birth or medical record number. To do this study, we will use the following kinds of PHI:

- Things you tell the researchers about your or your child’s health
- Information currently in your child’s medical records as well as information added to your child’s medical records during the course of this study.

## What happens if I say yes, but I change my mind later?

You can leave the research at any time. If you choose to leave the study, your choice will not affect you or your child’s healthcare or any services you receive. No matter what decision you make, and even if your decision changes, there will be no penalty to you. You and your child will not lose medical care or any legal rights.

If you stop being in the research, already collected data may not be removed from the study database. Your authorization for researchers to use you and your child’s protected health information (PHI) will last until the research study is done. However:

- You can choose to take back your authorization for researchers to use your health information. You can do this at any time before or during your participation in the research.
- If you take back your authorization, information that was already collected may still be used and shared with others, but the researchers will no longer be able to collect NEW information about you.
- If you take back your authorization, you will not be able to take part in the research study.
- To take back your authorization, you will need to tell the researchers by emailing the Lead Researcher, Dr. Michelle Kelly, at michelle.kelly@wisc.edu.

**Will being in this study help me in any way?**

By joining this study, you might get easier access to your child’s medical notes while they are in the hospital. This could help you understand their care better and talk with the hospital staff. Also, by being part of this study, you can help doctors and nurses learn more about how to support parents during their child’s hospital stay.

## What are the study risks?

The main risk of joining this study is that an unauthorized person might see the information we collect, but we have strong protections in place to prevent this. Another risk is that it could add stress for you, your family, or your child during the study.

## Who has access to the information collected for the research?

We have strict rules to protect your personal information and protected health information (PHI). We will limit the use and disclosure of your personal information, including research study and medical records, as described in this consent form.

However, we cannot promise complete confidentiality. We will share information with individuals or organizations identified in this consent form. Federal or state laws may also permit or require us to show information to university or government officials responsible for carrying out or monitoring this study. This includes the University of Wisconsin and its representatives and affiliates, including those responsible for ensuring compliance, such as the Human Research Protection Program.

The study is protected by a Certificate of Confidentiality from the Agency for Healthcare Research and Quality. This means we will not share any information that would identify you as a participant in the study, even if the police or courts ask to look at the data we have collected.

We may have to tell appropriate authorities, such as child protective services or health care providers, if we learn during the study that you or others are at risk of harm (for example, due to child or elder abuse, or suicidal thoughts).

Authorizing the research team to use your PHI means that we can release it to the people or groups listed in this form for the purposes described in this form. Once we share your identifiable health information outside UW-Madison, the HIPAA Privacy Rule may no longer protect it. However, we try to make sure that everyone who sees your health information keeps it confidential.

We will share information collected for this study with researchers or organizations outside UW-Madison, including study staff the other participating sites – Seattle Children’s Hospital and Children’s Hospital Los Angeles. A description of this clinical trial will be available on <http://www.ClinicalTrials.gov>. This Web site will not include information that can identify you. At most, the Web site will include a summary of the results. You can search this Web site at any time.

**Will information from this study go in my or my child’s medical record?**

None of the information we collect for this study will go in your or your child’s medical record.

## Will my or my child’s information be used in other research?

With appropriate confidentiality protections, we might use information that we collect during this study for other research or share it with other researchers without additional consent from you.

We may share survey responses or transcripts of interviews with researchers at other institutions. We will remove direct identifiers about you, such as you or your child’s name, date of birth and other information we think could be used to identify you or your child. However, we cannot guarantee there is no risk you or your child could be identified. Information in free text fields such as those in survey responses and transcripts of interviews may contain details that could be used with other available information to identify you (such as an employer name, job title or rare diagnosis).

We will share the data from this study in a database that collects information from research studies and is publicly available on a website. Anyone can use the data for any purpose in the future. This is called “open access.” We will remove any personal information that could identify you (like name, birthdate, age, gender, address, and medical record number). This is called de-identified data. We believe there is a low risk that de-identified study data could be used to re-identify you. However, it’s possible that data that cannot be used to identify you today could be used to identify you in the future.

## What else do I need to know?

Will I receive anything for participating?

If you participate in this study, you will receive a $20 gift card once both surveys are completed. If you do not complete the survey given at the time of your child’s discharge, you will not receive the $20 gift card. If you are selected for the optional interview, you will receive a second $20 gift card after the interview is completed. Not everyone will be selected for this interview.

## Permission to communicate about the study by email

We are requesting your email address so we can follow up with you if you are discharged before completing the discharge survey. Email is generally not a secure way to communicate about you or your child’s health as there are many ways for unauthorized users to access email. You should avoid sending sensitive, detailed personal information by email. Email should also not be used to convey information of an urgent nature. If you need to talk to someone immediately, please contact Dr. Michelle Kelly at 608-262-7229. You do not have to provide your email address to participate in this study.

## How many people will be in this study?

We expect about 630 people will be in this research study. This will consist of 600 parents and 30 hospital staff members at three participating sites.

**Who is funding/supporting this study?**

This research is being funded by the Agency for Healthcare Research and Quality.

**Agreement to participate in the research study**

| You are making a decision whether or not to participate in this study. You do not have to sign this form. If you refuse to sign, however, you cannot take part in this research study. If you sign the line below, it means that you have:   - read this consent and authorization form describing the research study procedures, risks and benefits - had a chance to ask questions about the research study and your participation, and received answers to your questions - decided to participate in this study - given authorization for the person’s protected health information to be used and shared as described in this form | | | | |
| --- | --- | --- | --- | --- |
| Signature of participant | |  | | Date |
|  | |  | | |
| Printed name of participant | |  |  |  |
|  | |  | |  |
| Signature of person obtaining consent | |  | | Date |
|  | |  | |  |
| Printed name of person obtaining consent | |  | |  |
|  |  | | | |
| Printed name of child |  |  |  |  |
|  |  | |  | |
| Typed name of parent or individual legally authorized to consent to the child’s general medical care |  | | Date | |
| - By checking this box and typing my name above, I am electronically signing this consent form. |  | |  | |
| **Note:** Investigators are to ensure that individuals who are not parents can demonstrate their legal authority to consent to the child’s general medical care. Contact legal counsel if any questions arise. | - Parent - Individual legally authorized to consent to the child’s general medical care (See note below) | | | |
|  |  |  |  |  |
|  | | | | |
