# Supplementary material for: A multicenter randomized trial to improve family clinical note access and outcomes for hospitalized children: The Bedside Notes study protocol
Source: J Hosp Med. 2025 Aug 21;20(11):1256–64. doi: 10.1002/jhm.70155 (PMC12443148; doi:10.1002/jhm.70155)
Supplement: Supplementary file 2 — Appendix 2 ‐ Enrollment Survey. [file JHM-20-1256-s002.docx]

Thank you for answering these questions about you and your hospitalized child. This information will help us understand how people from different backgrounds answer survey questions. Your responses will remain private.

**Instructions**: Please try to answer every question. If unsure, answer to the best of your ability or ask for clarification. It will take about 10 minutes to complete this survey.

*Si desea ver esta encuesta en español, haga clic en el símbolo del globo terráqueo que se encuentra arriba en la esquina derecha.*

**About your child and their health**

1. In general, how would you rate your child’s overall health?
   □ Excellent
   □ Very good
   □ Good
   □ Fair
   □ Poor
2. About how many times has your child ever been hospitalized?
   (Please do not count a routine birth hospitalization or their current hospital stay)

______ times

1. What is your child’s gender identity? *(Choose all that apply)*
   □ Female; Girl
   □ Male; Boy
   □ Non-binary
   □ Questioning, Exploring
   □ Prefer not to respond; Prefer not to disclose
   □ Gender identity not listed above → Please specify child gender: ___________________
2. What is your child’s assigned sex at birth?
   □ Female (F)
   □ Male (M)
   □ X
   □ Unsure
   □ Prefer not to respond; Prefer not to disclose
   □ Assigned sex not listed → Please specify child sex: ___________________
3. Please select the racial/ethnic category or categories your child most closely identifies with. *(Choose all that apply)*
   □ American Indian or Alaska Native
   □ Asian or Asian American
   □ Black or African American
   □ Hispanic, Latino, or Spanish
   □ Native Hawaiian or Other Pacific Islander
   □ Middle Eastern or North African
   □ White
   □ Other → Please specify child race: ___________________
   □ Prefer not to respond; Prefer not to disclose

□ Unknown

1. What language(s) do you and your family primarily speak at home?
   *(Choose all that apply)*
   □ English
   □ Spanish
   □ Other → Please specify other language ______________
2. Do the language(s) you listed above have a specific dialect that you prefer to use?

□ Yes → Please specify dialect: _____

□ No

1. What is your preferred language for study surveys?

□ English

□ Spanish

**About your role in managing your child’s health**

Please indicate how much you agree or disagree with each statement.

[Response options listed in survey: *Disagree strongly, Disagree, Agree, Agree strongly, N/A]*

1. When all is said and done, I am the person who is responsible for taking care of my child’s health.
2. Taking an active role in my child’s health care is the most important thing that affects their health.
3. I am confident that I can take actions to help prevent or reduce problems associated with my child’s health.
4. I know what each of my child’s prescribed medications do.
5. I am confident that I can tell whether I need to go to the doctor or whether I can take care of my child’s health problem myself.
6. I am confident I can tell a doctor the concerns that I have about my child’s health even when they do not ask.
7. I am confident that I can follow through on medical treatments I need to do for my child at home.
8. I understand my child’s health problems and what causes them.
9. I know what treatments are available for my child’s health problems.
10. I have been able to help my child maintain (keep up with) lifestyle changes, like eating right or exercising.
11. I know how to prevent problems with my child’s health.
12. I am confident I can figure out solutions when new problems arise with my child’s health.
13. I am confident that I can help my child maintain lifestyle changes, like eating right and exercising, even during times of stress.

We are also interested in how being in the hospital makes parents and caregivers feel.

Please indicate how you feel right now by choosing from the following options for each statement:

[Response options listed in survey: *Not at all, Somewhat, Moderately so, Very much so]*

1. I feel calm
2. I feel secure
3. I am tense
4. I feel strained
5. I feel at ease
6. I feel upset
7. I am presently worrying over possible misfortunes
8. I feel satisfied
9. I feel frightened
10. I feel comfortable
11. I feel self-confident
12. I feel nervous
13. I am jittery
14. I feel indecisive
15. I am relaxed
16. I feel content
17. I am worried
18. I feel confused
19. I feel steady
20. I feel pleasant

**About your language preferences and ability to communicate with your child’s medical care team**

1. Which language(s) do you want to use with your child’s medical care team?
   *(Choose all that apply)*
   □ English
   □ Spanish
   □ Other → Please specify other language: ______________
2. How comfortable are you with communicating in English with your child’s care team?

□ Very comfortable

□ Comfortable

□ Not comfortable

□ Not at all comfortable

□ Prefer not to respond; Prefer not to disclose

1. How often do you have someone (like a family member, friend, or hospital worker) help you read hospital materials?
   □ Always
   □ Often
   □ Sometimes
   □ Occasionally
   □ Never
2. How confident are you filling out medical forms by yourself?
   □ Extremely
   □ Quite a bit
   □ Somewhat
   □ A little bit
   □ Not at all
3. How often do you have problems learning about your or your child’s medical condition because of difficulty understanding written information?
   □ Always
   □ Often
   □ Sometimes
   □ Occasionally
   □ Never
4. How often do you have a problem understanding what is told to you about your or your child’s medical condition?
   *□* Always
   □ Often
   □ Sometimes
   □ Occasionally
   □ Never

**About your experience accessing your child’s medical information online using a patient portal, like MyChart**

1. How important would it be for you to get your child’s medical information online on a patient portal, like MyChart?
   □ Very important
   □ Somewhat important
   □ Not at all important
2. Have you ever been offered online access to your child’s medical information by a health care provider before this hospital stay?
   □ Yes
   □ No
   □ Don’t know
3. Have you ever used a patient portal, like MyChart, to access your child’s medical information online before this hospital stay?
   □ Yes
   □ No
   □ Don’t know

*If no or don’t know skip next three questions, if yes, continue*

1. What did you access on the patient portal? *(Choose all that apply)*
   □ Test results
   □ Medications
   □ Notes
   □ Secure messages to the care team
   □ Virtual visits
   □ Appointment scheduling
   □ Bill payment
   □ Other → Please specify other access on patient portal: ___________________
2. What technology did you use to access the portal? *(Choose all that apply)*
   □Cell phone
   □ Tablet

□ Laptop computer

□ Desktop computer
□ Other → Please specify other technology: ___________________

1. How many times have you used a patient portal to access your child’s medical information in the last 12 months?
   □ None
   □ 1 to 2 times
   □ 3 to 5 times
   □ 6 to 9 times
   □ 10 or more times

**About you**

1. What is your age?
   □ 18-24
   □ 25-34
   □ 35-44
   □ 45-54
   □ 55-64
   □ 65 and older
2. How are you related to the child?
   □ Mother
   □ Father
   □ Other relative or legal guardian → Please specify other role: ___________________
3. What is your current gender identity? *(Choose all the apply)*

□ Man

□ Woman

□ Non-Binary

□ Transgender

□ None of these describe me. I would like to consider additional options.

→ Please specify other gender: _______________________

□ Prefer not to answer

1. What was your biological sex assigned at birth (on your original birth certificate)?

□ Female

□ Male

□ Intersex

□ None of these describe me → Please specify other sex: _______________________

□ Prefer not to answer

1. Please select the racial/ethnic category or categories with which you most closely identify with. *(Choose all the apply)*

□ American Indian or Alaska Native

□ Asian or Asian American

□ Black or African American

□ Hispanic, Latino, or Spanish

□ Native Hawaiian or Other Pacific Islander

□ Middle Eastern or North African

□ White

□ Other → Please specify other race: ______________

□ Prefer not to respond; Prefer not to disclose

1. What is the highest grade or level of school you have finished?
   □ Primary school to 8th grade, or less
   □ Some high school
   □ High school graduate or high school equivalency diploma (GED)
   □ Some college or 2-year college degree (e.g., trade or technical school)
   □ 4-year college graduate

□ Master’s program graduate

□ Doctoral program graduate

□ Education level not listed above → Please specify other education: ____________

□ Prefer not to respond; Prefer not to disclose

1. Now we would like to ask about how much money your family earns in a year. We know this is a private issue, but it is important because your income affects your health and how you and your family get healthcare.
   Please look at this list and pick the group that is closest to your total household income in the past 12 months.
   □ Less than $14,999
   □ Between $15,000 and $19,999
   □ Between $20,000 and $29,999
   □ Between $30,000 and $49,999
   □ Between $50,000 and $74,999
   □ Between $75,000 and $99,999
   □ Between $100,000 and $149,999
   □ Greater than $150,000
   □ Prefer not to answer
2. Including (counting) yourself, how many people live on this income? _________ people

We will ask you to complete a second survey around the time of your child’s discharge. It’s possible you’ll go home before we get the chance to give you the second survey. In that case, we’ll send you the survey to complete remotely.

1. What is your phone number? (optional) ________________
2. What is your email address? (optional) ______________
3. Please select how you would prefer study staff to contact you (choose all that apply):

□ By text message

□ By phone call

□ By email

□ I do not want study staff to contact me.

Thank you for completing this survey! Your responses will help us improve care for families like yours. If you have questions, contact Michelle Kelly: michelle.kelly@wisc.edu.
