# Supplementary material for: A multicenter randomized trial to improve family clinical note access and outcomes for hospitalized children: The Bedside Notes study protocol
Source: J Hosp Med. 2025 Aug 21;20(11):1256–64. doi: 10.1002/jhm.70155 (PMC12443148; doi:10.1002/jhm.70155)
Supplement: Supplementary file 3 — Appendix 3 ‐ Discharge Survey. [file JHM-20-1256-s001.docx]

Thank you for answering these questions about you and your hospitalized child. This information will help us understand how people from different backgrounds answer survey questions. Your responses will remain private.

**Instructions**: Please try to answer every question. If unsure, answer to the best of your ability or ask for clarification. It will take about 10 minutes to complete this survey

*Si desea ver esta encuesta en español, haga clic en el símbolo del globo terráqueo que se encuentra arriba en la esquina derecha.*

**About your experience accessing your child’s medical notes during this hospital stay**

1. Were you aware that you could access medical notes written about your child during this hospital stay?

□ Yes

□ No

□ Other → *Please specify other: _______________*

*If No, skip to question 10*

1. About how many of your child’s medical notes did you access during this hospital stay?
   □ None
   □ One note
   □ 2-5 notes
   □ 6-10 notes
   □ 11-20 notes
   □ Over 20 notes

*If None, skip to question 10*

1. On a scale of 1 - 5, how well did you understand your child’s medical notes?
   □ 1 l did not understand them at all
   □ 2
   □ 3
   □ 4
   □ 5 Understood them completely
2. On a scale of 1 - 5, how well did your child’s medical notes describe what happened during this hospital stay?
   □ 1 Not well at all
   □ 2
   □ 3
   □ 4
   □ 5 Extremely well
   □ Don’t know
3. Did you notice anything in your child’s medical notes that may be inaccurate, aside from misspellings and typing errors? (For example, inaccurate medicines, medical problems, family medical history, or past surgeries)
   □ Yes
   □ No
   □ I am not sure

*If No, skip to question 10*

1. About how many possible inaccuracies did you notice? *(Choose one*)

□ 1

□ 2

□ 3

□ 4

□ 5

□ 5 or more

*Repeat 7-9 for each inaccuracy [indicated in Q6, up to 5 inaccuracies]*

*Please tell us about the first error:*

1. What kind of possible inaccuracy did you find? *(Choose one)*
   □ Medicines my child takes → *Please specify medicine error: __________________________*
   □ What brought my child to the hospital
   □ My child’s other health problems
   □ My family’s medical history
   □ My child’s physical exam
   □ Updates from overnight
   □ The assessment of my child’s medical problems
   □ My child’s plan for the day
   □ My child’s vaccination history
   □ Names of my child’s health care providers

□ Something important was missing from my child’s note(s)
□ Other → *Please specify other error: ________________________*

1. Please tell us more about the inaccuracy you found. *(Free text)*
2. On a scale of 1 - 5, how important do you think this inaccuracy was?
   □ 1 Not at all important
   □ 2
   □ 3
   □ 4
   □ 5 Very important

**About your experience in the hospital**

1. During this hospital stay, how often did your child’s **doctors** give you as much information as you wanted about your child’s plan for the day?
   □ Never
   □ Sometimes
   □ Usually
   □ Always
2. During this hospital stay, how often did your child’s **nurses** give you as much information as you wanted about your child’s plan for the day?
   □ Never
   □ Sometimes
   □ Usually
   □ Always
3. During this hospital stay, how often did your child’s **doctors** listen carefully to you?
   □ Never
   □ Sometimes
   □ Usually
   □ Always
4. During this hospital stay, how often did your child’s **nurses** listen carefully to you?
   □ Never
   □ Sometimes
   □ Usually
   □ Always
5. During this hospital stay, how often did your child’s **doctors** treat you with courtesy and respect?
   □ Never
   □ Sometimes
   □ Usually
   □ Always
6. During this hospital stay, how often did your child’s **nurses** treat you with courtesy and respect?
   □ Never
   □ Sometimes
   □ Usually
   □ Always
7. Did you trust the **doctors** treating your child?
   □ No
   □ Yes, sometimes
   □ Yes, always
8. Did you trust the **nurses** treating your child?

□ No
□ Yes, sometimes
□ Yes, always

1. Is there anything else you’d like to report about your interactions with staff members at this hospital?

□ No

□ Yes → Please Describe: ________

1. Mistakes in your child’s health care can include things like giving the wrong medicine or doing the wrong surgery. During this hospital stay, did providers or other hospital staff tell you how to report if you had any concerns about mistakes in your child’s health care?

□ No
□ Yes, sometimes
□ Yes, definitely

1. Using any number from 0 to 10, where 0 is the worst hospital possible and 10 is the best hospital possible, what number would you use to rate this hospital during your child’s stay?

□ 0 Worst hospital possible
□ 1
□ 2
□ 3
□ 4
□ 5
□ 6
□ 7
□ 8
□ 9
□ 10 Best hospital possible

1. Would you recommend this hospital to your friends and family?

□ Definitely no
□ Probably no
□ Probably yes

□ Definitely yes

**About your understanding of your child’s hospital care**

Please indicate how much you agree or disagree with each statement below: *(Response options listed in survey: Strongly Disagree, Disagree, Neither Agree nor Disagree, Agree, Strongly Agree)*

| 1. I understand the reason why my child was admitted to the hospital. |
| --- |
| 1. I understood my child’s plan of care each day in the hospital. |
| 1. I understand my child’s goals for discharge from the hospital. |

**About your role in managing your child’s health**

Please indicate how much you agree or disagree with each statement. *(Response options listed in survey: Disagree strongly, Disagree, Agree, Agree strongly, N/A*

1. When all is said and done, I am the person who is responsible for taking care of my child’s health.
2. Taking an active role in my child’s health care is the most important thing that affects their health.
3. I am confident that I can take actions to help prevent or reduce problems associated with my child’s health.
4. I know what each of my child’s prescribed medications do.
5. I am confident that I can tell whether I need to go to the doctor or whether I can take care of my child’s health problem myself.
6. I am confident I can tell a doctor the concerns that I have about my child’s health, even when they do not ask.
7. I am confident that I can follow through on medical treatments I need to do for my child at home.
8. I understand my child’s health problems and what causes them.
9. I know what treatments are available for my child’s health problems.
10. I have been able to help my child maintain (keep up with) lifestyle changes, like eating right or exercising.
11. I know how to prevent problems with my child’s health.
12. I am confident I can figure out solutions when new problems arise with my child’s health.
13. I am confident that I can help my child maintain lifestyle changes, like eating right and exercising, even during times of stress.

We are also interested in how being in the hospital makes parents and caregivers feel. Please indicate how you feel right now by choosing from the following options for each statement:

1. I feel calm
2. I feel secure
3. I am tense
4. I feel strained
5. I feel at ease
6. I feel upset
7. I am presently worrying over possible misfortunes
8. I feel satisfied
9. I feel frightened
10. I feel comfortable
11. I feel self-confident
12. I feel nervous
13. I am jittery
14. I feel indecisive
15. I am relaxed
16. I feel content
17. I am worried
18. I feel confused
19. I feel steady
20. I feel pleasant

Thank you for completing this survey! Your responses will help us improve care for families like yours. If you have questions, contact Michelle Kelly: michelle.kelly@wisc.edu.
